# Placental Pathology in Obstetric Antiphospholipid Syndrome Beyond Thrombosis: A Case Report and Literature Review

**DOI:** 10.3390/jcm14155172

**Published:** 2025-07-22

**Authors:** Dagmara Dzirba, Malwina Glinko, Marta Skoczyńska, Katarzyna Gruszecka, Martyna Trzeszcz, Adam Benedyczak, Magdalena Szmyrka

**Affiliations:** 1 Lower Silesian Oncology Center, 53-413 Wroclaw, Poland; malwina.glinko@gmail.com; 2 Department of Rheumatology and Internal Medicine, Lower Silesian Specialist Hospital, 54-049 Wroclaw, Poland; marta.skoczynska@gmail.com (M.S.); katarzyna.gruszecka@pwr.edu.pl (K.G.); 3 Department of Non-Procedural Clinical Sciences, Faculty of Medicine, University of Technology and Science, 58-376 Wroclaw, Poland; 4 Department of Pathology and Clinical Cytology, University Clinical Hospital, 50-556 Wroclaw, Poland; m.trzeszcz@corfamed.pl; 5 4th Military Clinical Hospital, 50-981 Wroclaw, Poland; adam_benedyczak@wp.pl; 6 Department of Rheumatology and Internal Diseases, Faculty of Medicine, Wroclaw Medical University, 50-556 Wroclaw, Poland; magdalenaszmyrka@gmail.com

**Keywords:** obstetric antiphospholipid syndrome, placental insufficiency, placental pathology, pregnancy monitoring

## Abstract

**Background**: Antiphospholipid syndrome (APS) is one of the highest risk factors for obstetric complications. This article contains a case report of a patient with obstetric APS who experienced fetal loss during their first pregnancy and experienced a successful second pregnancy upon treatment with acetylsalicylic acid (ASA), low-molecular-weight heparin (LMWH), and hydroxychloroquine (HCQ). We compare placental pathology in these two pregnancies and discuss the impact of antiphospholipid antibodies and clinical management on pregnancy outcomes. We also propose methods to monitor obstetric antiphospholipid syndrome (OAPS) patients during pregnancy. **Methods**: A 26-year-old woman presented with a history of stillbirth at 25 weeks of pregnancy due to placental insufficiency. Before pregnancy, she experienced symptoms suggestive of autoimmune disease (thrombocytopenia, recurrent mouth aphthous ulcers, and Raynaud’s phenomenon) but had no diagnosis. Placental dysfunction correlated with the high ratio of sFlt-1/PIGF (soluble fms-like tyrosine kinase 1 and the placental growth factors index). Laboratory tests revealed the presence of antinuclear antibodies (ANAs) and triple positivity for antiphospholipid antibodies (aPLs). **Results**: Following the initiation of treatment for OAPS and regular monitoring consistent with current guidelines, the patient conceived and successfully delivered a healthy child. **Conclusions**: Adequate therapy and close monitoring during pregnancy, including clinical observation, placental biomarkers and regular ultrasonography, may help to reduce the risks and increase chances for optimal pregnancy outcomes. Additionally, pathological examination and clinical collaboration are essential components in future pregnancy counseling and should be a part of multidisciplinary management.

## 1. Introduction

Antiphospholipid syndrome (APS) is an autoimmune disorder that affects both large and small blood vessels. Clinical manifestations may include venous thromboembolism (which is present in approximately 30–40% of cases with deep vein thrombosis as the most common manifestation), arterial thrombosis (observed in approximately 20–30% of cases, including strokes in 19.8% of cases and myocardial infarctions in 5.5% of cases), and obstetric complications [1]. According to the new classification, APS obstetric complications include the following [2]:

- A total of ≥3 consecutive pregnancy losses that occur either before 10 weeks (pre-fetal) or between 10 weeks and 15 weeks from 6 days (early fetal).
- The death of the fetus between 16 weeks 0 days and 33 weeks from 6 days in the absence of preeclampsia with severe features or placental insufficiency with severe features.

Each complication is given a weight of one.

- Preeclampsia and/or placental insufficiency with severe features occurring before 34 weeks from 0 days with or without fetal death with an assigned respective weight of four and three.

Obstetric complications are typically the first symptoms of APS observed in women and pose a threat to both mother and fetus. In pregnant women with APS specifically, pregnancy loss (22.2%) and fetal growth restriction (FGR) (25.9%) are observed more frequently [3]. According to an EUROAPS report from 2019 that included a cohort of 1000 obstetric APS patients, miscarriages were the most common complication (in 38.6% of cases), followed by early preeclampsia (PE) (18.1%) and early FGR (16.1%) [4].

Approximately 6% of women experiencing pregnancy loss, FGR, PE, or eclampsia test positive for antiphospholipid antibodies (aPLs) [5]. Taking into account all aPLs, the worst obstetric predictor after the first trimester is the presence of a lupus anticoagulant (LA) [6]. Although the meta-analysis from 2022 excluded a significant relationship between LA and FGR, pointing to anticardiolipin antibodies (ACAs) and anti-beta 2 glycoprotein 1 (β2GP1) involvement [7], the risk of early pregnancy loss is especially high in the presence of LA accompanied by positive ACAs [8]. The detection of these two kinds of antibodies is also associated with PE [9].

The etiology of APS has not been completely understood, but its causes have been linked to factors such as the genetic predisposition (the role of human leukocyte antigens (HLAs) of class II molecules [10] and amino acid polymorphism changes at a specific position (valine to leucine replacement) in the structure of the potential epitope for aβ2GPI [11], which induce complement-involved processes leading to placental pathologies, especially in recurrent pregnancy loss, as shown in ref. [12]. Known triggers are also infections. There is an observed correlation between ACA and HIV, HCV, HBV, EBV, and HTLV-1 and between aβ2GPI and HCV [13]. Bacterial and virus infections can intensify inflammatory and thrombotic cascade, leading to multiorgan thrombosis known as a catastrophic APS (CAPS), where 80% of cases are linked to pregnancy [14,15]. Additionally, Baños et al. indicate a correlation between demographic characteristics such as non-Caucasian ethnicity, smoking, and adverse pregnancy outcomes [16]. In APS, having a history of thrombosis is a significant risk factor associated with a decreased live birth rate, increased risk of neonatal mortality, both prenatal and postnatal thrombosis, and a higher likelihood of delivering small-for-gestational-age (SGA) neonates. Moreover, the double or triple positivity of aPLs correlates with decreased live birth rates and an increased risk of SGA neonates, preterm birth, and PE. The two previous complications are significantly linked to the presence of LA [17].

## 2. Case Report

A 26-year-old woman presented to the rheumatology clinic in February 2024 with a history of fetal loss during her first pregnancy and suspicion of connective tissue disease. In July 2023, as a result of placental insufficiency, the patient experienced stillbirth at 25 weeks of her first pregnancy. Prior to the pregnancy loss, the fetus was diagnosed with stage III FGR. Sample material obtained from amniocentesis was genetically tested, revealing no signs of karyotype abnormalities.

Placental dysfunction correlated with the high sFlt-1/PIGF index value of 468. The placental examination was performed by a qualified perinatal pathologist according to the protocol and diagnostic criteria of the Amsterdam Placental Workshop Group Consensus Statement [18].

In a rheumatology consultation on 02.2024, the patient complained of chronic fatigue, recurrent mouth aphthous ulcers, Raynaud’s symptoms, and alopecia in the forehead area. Looking at her past history, in 2020, she was diagnosed with hypothyroidism and thrombocytopenia and treated with prednisone at 70 mg p698–713er day for a month with gradual dose tapering. The diagnostic evaluation was not extended to include rheumatologic diseases until 2024. On 02.2024, she tested positive for aPLs (triple positive: LA, ACA-390.9 U/mL and aβ2GPI-1462 U/mL class IgG (normal range 0–20 U/mL)) and ANA (1:10,000 (normal range < 1:80). The APLs were tested again 12 weeks later and revealed triple positivity again.

These test results lead to the diagnosis of obstetric APS and undifferentiated connective tissue disease—most probably lupus-like disease. She received treatment with hydroxychloroquine (200 mg/d) and low-dose aspirin (LDA) (75 mg/d) and was informed that in case of future pregnancies, she should receive treatment with LMWH.

With these points in mind, we reviewed the results of placental pathology in failed pregnancy. Due to abnormal lesions identified by gross examination, the following additional samples were submitted: (a) 2 samples of the extraplacental membranes; (b) 4 cross-sections of the umbilical cord (2 for the fetal end and 2 for the placental end); and (c) 15 samples from the placental parenchyma, including 12 from abnormal areas and 3 full-thickness samples from normal areas.

**Figure 1:**
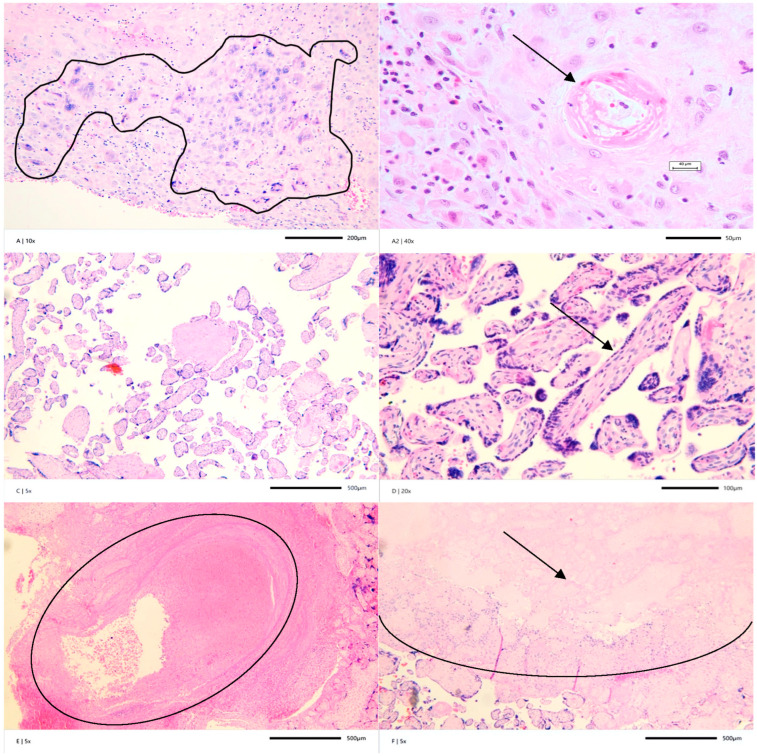
Placental pathology. (**A**) The presence of giant trophoblastic cells in the basal decidua (highlighted field). This feature lies in a spectrum of the developmental abnormalities of the maternal stromal-vascular compartment of the placenta, H&E-stained section at ×10. (**A2**) Decidual arteriopathy: atherosclerosis in the spiral arteries (arrow) of the basal plate, H&E-stained section at ×40. (**C**) Distal villous hypoplasia: the predominance of villous paucity is seen due to decreased villous branching, H&E-stained section at ×5. (**D**) A centrally located longitudinal profile of small caliber filiform villi (arrow) in distal villous hypoplasia, H&E-stained section at ×20. (**E**) The retroplacental microhematoma (area circled), H&E-stained section at ×5. (**F**) One of numerous (15) infarcts found (demarcated area), H&E-stained section at ×5.

It should be noted that the placental examination showed 15 white-yellowish infarcts involving nearly 70% of the placental parenchyma, with the largest lesion being 1.5 cm in diameter (Figure 1F). The retroplacental hematoma was not identified grossly (with an involvement below 5% of the placenta, with the largest dimension being 1.0 cm) (Figure 1E). The fixed placenta weight was 105 g (below the fifth percentile at 25 weeks of gestation) with paracentric cord insertion. The fetal–placental weight ratio was over the 90th percentile: 5.2). The atherosclerosis of spiral arteries in the basal plate presented intraluminally and was seen upon microscopic examination, the findings of which are consistent with decidual arteriopathy (Figure 1A). The presence of giant trophoblastic cells in basal decidua (Figure 1A2) is consistent with shallow placental implantation. Terminal villous hypoplasia with long, filiform, unbranched villi, decreased syncytial knotting, and a wide intervillous space is shown in Figure 1C,D. All microimages in this case were acquired using the EPview camera, version V3.7.7_20230531 (Olympus, Evident Technology Center Europe, Warsaw, Poland) in a 300 dpi resolution.

In May 2024, the patient became pregnant again. She was treated with hydroxychloroquine at an increased dose of 400 mg/day, ASA at a dose of 150 mg/day, and LMWH was introduced at a prophylactic dose (40 mg/day) with thyroid hormones. CBC (complete blood count) showed WBCs of 7120, Hb levels of 13.1 g/dL, and PLT levels of 341,000. ANA was positive at 1:3200 with anti-RNP. The pregnancy was not complicated. A male infant was delivered in January 2025 at 38 weeks of gestation via cesarean section, with an Apgar score of 10 and a birth weight of 2870 g (16th percentile). A gross placental examination after the second pregnancy revealed a small placenta for gestational age: the fixed placenta weight was 355 g (below the 10th percentile at 38 weeks of gestation). The fetal–placental weight ratio was over the 90th percentile: 8.44. The umbilical cord index was over the 90th percentile (UCI: 0.63), which is consistent with a hyper-coiled umbilical cord. Placental parenchyma showed a single infarct involving below 5% of the placental tissue, 0.5 cm in diameter. The microscopic evaluation showed small foci of karyorrhectic villi consistent with low-grade segmental fetal vascular malperfusion, probably secondary to the hyper-coiling of the umbilical cord identified. The presence of giant trophoblastic cells in basal decidua was associated with developmental abnormalities in the maternal stromal-vascular compartment, including the shallow implantation of the placenta. The placental examination did not reveal lesions indicative of severe placental insufficiency, as observed in the patient’s previous pregnancy, which resulted from vascular developmental abnormalities and a causally linked cascade—decidual arteriopathy, early global maternal malperfusion, and multiple infarcts involving 70% of the placental parenchyma. However, it should be noted that an abnormal fetoplacental ratio and placental hypoplasia are surrogate markers of placental function, and in this case—during the second pregnancy—they were abnormal.

The patient stayed under the collaborative care of a gynecologist–obstetrician and rheumatologist.

**Figure 2:**
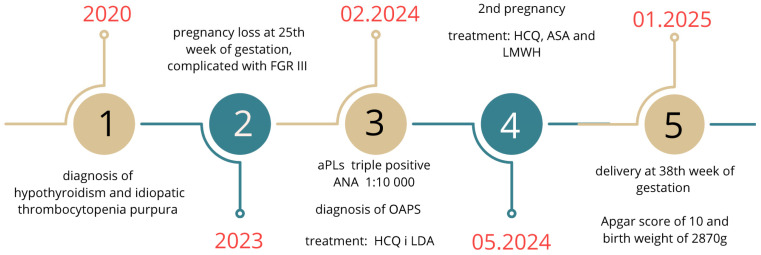
Timeline of the Patient’s Medical History.

During the rheumatological follow-up in May 2025, the patient was in a good general state and did not have any new manifestations of autoimmune diseases; the results of her laboratory tests did not show any abnormalities, especially thrombocytopenia and abnormalities in clotting times. The patient is still under rheumatological follow-up, continuing treatment with HCQ 200 mg/d and ASA 75 mg/d. She currently does not meet the criteria for systemic lupus erythematosus (SLE) or any other connective tissue disease (CTD) (Figure 2).

## 3. Discussion

In 2023, new, highly sensitive APS classification criteria were published. In the new set of APS criteria, clinical symptoms unrelated to thrombotic events and not included in the 2006 classification criteria are taken into account [2]. The latest ACR/EULAR 2023 classification criteria for APS includes the following points [2] (Table 1):

The new criteria of APS are assigned a certain number of points, and in obstetric criteria, preeclampsia with severe features or/and placental insufficiency with severe features are regarded as more specific for OAPS than recurrent early fetal loss (Table 2).

Although, for many years, APS was regarded as primarily caused by thrombosis, nowadays, there is data proving that it is not the only mechanism, especially in the obstetric variant of the disease. The complications in obstetric APS are believed to be primarily due to abnormal trophoblast implantation, the impaired remodeling of the uteroplacental arteries, inflammation in the maternal–fetal space, an increased number of syncytial nuclear aggregates (SNAs), and reduced vasculosyncytial membranes (VSMs) [21].

Noteworthy, thrombotic events within the placental parenchyma or fetal placental vessels are not a common cause of obstetric complications in APS patients [22,23,24]. These findings were also present in this case report, where placental histopathology revealed severe placental insufficiency caused by severe maternal vascular malperfusion (MVM) associated with the abnormal remodeling of spiral arteries (features of decidual arteriopathy), with no accompanying evidence of fetal vascular malperfusion (FVM).

In optimal conditions, the trophoblastic invasion of decidua takes place during the first trimester of pregnancy, and spiral arteries are the target for extravillous trophoblasts (EVTs). EVTs pass through musculo-elastic layers and replace epithelial cells. This link acts as a gateway for blood plasma containing aPLs, allowing them to move freely among loosely structured trophoblast plugs [25,26].

In pathological conditions, spiral arteries are only partially remodeled, which leads to impeded blood transport across the placenta [27]. During the invasion stage, the trophoblast exposes phospholipid anions, and phospholipid-binding proteins (β2G PI) attach to them, becoming targets for aPLs [28,29]. The aPL-β2G PI complex interacts with toll-like receptor 4 (TLR-4), apolipoprotein E receptor 2 (ApoER2), and endothelial protein C receptor (EPCR) located on the surface of target cells, resulting in the activation of inflammatory pathway mediated by interleukin-8 (IL-8), interleukin-6 (IL-6), tumor necrosis factor α (TNF-alpha), and interferon alpha (IFN-alpha) cytokines. These target cells are trophoblasts, epithelial and endometrial stroma cells, as well as monocytes and neutrophils from the maternal–fetal space [25,30,31,32]. All these processes may impede the proliferation and invasion of EVTs and consequently lower the production of HCG (human chorionic gonadotropin) along with proangiogenic factors [33,34,35,36,37,38].

The reduction in blood supply to the placenta results in placental hypoxia and ischemia. The resulting impaired transport of nutrients, coupled with an increase in blood flow speed, ultimately leads to placenta damage [25,27].

Focusing on the binding points of aPLs associated with functional damage on placental tissue, several distinct pathways are highlighted, which help explain the multiplicity and diversity of clinical manifestations in OAPS [39]. Antibodies can interfere with annexin V, which provides a thromboregulatory function and maintains placental integrity [31,40,41].

Moreover, aPLs impair trophoblast invasiveness by inhibiting the enzymes (metalloproteases-2 and -9), causing the extracellular degradation of that process [42,43,44,45].

In mice with aPLs, a histological examination of the placenta revealed decidual necrosis with IgG and fibrin deposition, alongside complement-mediated inflammation within decidual cells marked by the accumulation of C3 and C5 [46]. A proinflammatory state induced by antibodies can also be facilitated by neutrophil infiltration and the local secretion of TNF-alpha [47].

However, complement activation causes endothelial injury and disrupts vascular integrity, predisposing the patient to a prothrombotic state consistent with the ‘two hit” pattern [48]. Finally, impaired blood flow leads to placental dysfunction, which manifests as stillbirth, FGR, or PE [49,50].

The pathomechanism described likely occurred in our case. Maternal developmental lesions in the placenta, manifesting as decidual arteriopathy, reflect a lack of physiological conversion of the spiral arteries in the basal plate. Under these arteriopathic conditions, early MVM developed, characterized by terminal villous hypoplasia, which is strongly associated with FGR and abnormal Doppler in umbilical arteries. Subsequently, multiple placental parenchymal infarcts formed, occupying an extensive placental area (70%). The identified retroplacental microhematoma was likely associated with severe damage to the spiral arteries. The placental injury was significant, as evidenced by significant placental hypoplasia (weight < 5th percentile) and an abnormal fetal–placental index above the 90th percentile, indicating a compromised functional placental reserve. As demonstrated in our case, in the placentas of mothers with APS, pathologic features can be morphologically indistinguishable from those presented in women with maternal hypertensive disease, thrombophilia, systemic lupus erythematosus, or APS [51]. An Italian cohort study examined the placentas of pregnant APS patients, both primary and secondary to SLE. The findings revealed that 68% had increased syncytial knots, 44% showed decreased vasculosyncytial membranes, and infarctions were observed in 32% of placentas examined, all despite treatment. Impaired spiral artery and decidual inflammation were also found. Moreover, placentas with syncytial knots and at least two types of lesions correlated with the higher titers of ACA IgG. Placental infarctions were observed in all patients with thromboembolic history, leading to lower placental and neonatal weights [52].

In a broader context, a prospective study collected placentas from pregnant women with autoimmune diseases (AIDs) such as SLE, APS, and non-criteria obstetric APS (NC-OAPS), searching for different patterns of histopathological findings [53]. The results were as follows: the lowest mean values for placental weight occurred in the NC-OAPS and significantly in the APS group. In addition, APS placentas were marked by significant maternal-side malperfusion; however, fetal-side maldevelopment was observed more often in NC-OAPS placentas. In this study, placenta pathologies such as maternal malperfusion and fetal maldevelopment clearly correlated with an increased frequency of adverse perinatal outcomes [53].

The consequence of placental malperfusion is the production of sFlt-1 and soluble endoglin. sFlt-1, through VEGF (vascular endothelial growth factor) and PlGF sequestering, contributes to the inhibition of angiogenesis within the placenta [54,55,56].

These mechanisms contribute to the high risk of preeclampsia in pregnant women with APS [57]. The method of detecting placental damage associated with preeclampsia involves the measurement of the sFlt-1/PlGF index from a blood sample. Significantly increased levels of endoglin and sFlt-1, as well as an increased sFlt1/PlGF index and decreased levels of PlGF, observed from the 12th week of gestation in women with APS and/or SLE, correspond with the risk of preeclampsia [58]. The study conducted by Mayer-Pickel et al. identified 26% of women (diagnosed with APS and SLE) who developed preeclampsia later on and proved the clinical utility of measuring angiogenic factors levels such as endoglin, sFlt-1, and PlGF. This study highlights the importance of the regular monthly monitoring of the sFlt-1/PlGF ratio in high-risk pregnancies in particular [59]. The correlation between placental pathology and biochemical placental biomarkers has become an important aim of research. A retrospective analysis of 328 patients comparing PE, IUGR, PE + IUGR, and control groups revealed a negative correlation between the sFlt-1/PlGF ratio and placental mass; this was most evident in the PE + IUGR group (and the least evident in controls) [60]. Furthermore, a prospective cohort study conducted as part of the POP Study project evaluated the prognostic value of the sFlt-1/PlGF ratio in IUGR, showing a significant correlation with fetal morphometry and markers of placental dysfunction [61]. Accordingly, the results of a cohort study identified the association and clinical utility of fetoplacental Doppler parameters (UtA-PI (uterine artery pulsatility index) > 95th centile and CPR (cerebroplacental ratio) < 5th centile) with placental pathology, particularly MVM. These findings highlight placental biomarkers as a prognostic prenatal indicator of FGR [62]. Real-world clinical data from such retrospective single-center studies confirm a positive correlation between the sFlt-1/PlGF ratio and placental dysfunction and an inverse association with gestational age at delivery or birth weight [63].

In our study, placental examination clearly correlated with abnormal sFlt/PlGF ratio maternal serum levels. Thus, we believe that abnormal biochemical placental biomarkers in the mother could serve as a valuable additional indication for the pathological examination of the placenta. This could also be important for future clinical research aimed at optimizing both the selection of diagnostic methods and the interpretation of results, ultimately influencing future pregnancy counseling.

Placental abnormalities lead to FGR. The diagnosis of this is currently based on the Delphi procedure and consensus definition of FGR. Congenital anomalies, TORCH infections (an acronym for toxoplasmosis and other infections such as rubella, cytomegalovirus, and herpes simplex virus), and chromosomal abnormalities must be excluded during the diagnostic process [64].

The presence of at least one major risk factor (or three minor such as previous preeclampsia, primiparity, or time between pregnancies < 6 months) significantly increases the risk of developing FGR [65]. APS (with one of the highest relative risks of 6.2), diabetes with vascular complications, renal failure, or severe pregnancy-induced hypertension are recognized as major risk factors.

The recommendations of the American College of Obstetricians and Gynecologists (ACOG) and the International Society for the Study of Hypertension (ISSHP) advise conducting screening from the first trimester onwards for PE in women with maternal risk factors indicating autoimmune diseases [66]. In a group at high risk of developing FGR/preeclampsia, additional ultrasound, and laboratory screenings should be considered between 19 and 24 weeks of gestation on the basis of medical history and UtA, PI, mean arterial pressure (MAP), PlGF, and sFlt-1 levels [64].

The PROMISSE Study (Predictors of pregnancy outcome: biomarkers in antiphospholipid antibody syndrome and systemic lupus erythematosus) also indicates other biomarkers for placental insufficiency and pregnancy complications in pregnant women with APS. The research findings show that elevated levels of complement activation fragment, modest increases in C3, and notably elevated serum uric acid levels (in mid-gestation) are associated with adverse pregnancy outcomes [25,67,68].

## 4. Therapy in OAPS

### 4.1. Standard Treatment

The primary preventive strategy in obstetric APS complications involves the introduction of LDA during the preconception period. Early intervention is associated with better reproductive effects than the use of LDA after pregnancy confirmation. Aspirin improves the physiological transformation of spiral uterine arteries and rheological conditions in the placenta. LDA is recommended, especially in women with high-risk aPL profiles [69,70]. The standard obstetric treatment of OAPS also includes heparin: LMWH or unfractionated heparin (UFH) in prophylactic doses. Heparin should be introduced immediately after the confirmation of pregnancy in patients with previous complications. Patients should continue antithrombotic therapy until 6–12 weeks after the end of pregnancy due to the increased risk of thrombosis in the postpartum period [69,71]. Approximately 20–30% of women with APS receiving both LDA and heparin are still unable to maintain their pregnancy and deliver a healthy child [72,73]. Comprehensive methods of treatment and management in OAPS are presented in Table 3, including previous obstetric complications, thrombotic events, non-criteria manifestations, with or without the presence of aPLs, and subsequent lines of therapy.

### 4.2. Immunomodulatory Treatment

A more recent approach to obstetric APS treatment, recognizing the role of inflammation in APS obstetric complications, includes immunomodulatory therapy with hydroxychloroquine, low-dose steroids, immunoglobulin (IVIG), or plasmapheresis [69,71].

Hydroxychloroquine increases the pH in lysosomes, reducing T-cell activation, inhibiting proinflammatory cytokines, and modulating innate immune responses. In OAPS, HCQ has therapeutic potential by inhibiting complement activation and tissue factor (TF) production, disrupting the formation of immune complexes (e.g., aPL–antigen binding), and reducing endothelial dysfunction and oxidative stress (e.g., via NOX2 inhibition) [76]. Ruffatti et al. (2018) propose that hydroxychloroquine can be used in APS refractory cases as well as conventional medications in APS and patients with triple aPL positivity (LA, ACA, and aβ2-GPI) [77]. The addition of HCQ improves live birth rates and reduces pregnancy complications, especially when treatment begins with preconception at 400 mg daily. While HCQ is generally considered safe during pregnancy, recent data suggest a slightly increased risk of congenital anomalies (e.g., urinary tract or oral clefts) with first-trimester exposure at higher doses (≥400 mg/day) [78]. The HYPATIA ongoing trial (HYdroxychloroquine to improve Pregnancy outcome in women with AnTIphospholipid Antibodies) aims to better define the efficacy and safety of HCQ in pregnancy for APS patients [79].

EULAR recommendations also suggest combined therapy with glucocorticoids (GCSs) for first-line treatment in CAPS [69]. Nowadays, studies have shown that properly managed GCS provides therapeutic efficacy for both maternal and fetal health along with HCQ and LDA [80].

### 4.3. Refractory Cases

Intravenous immunoglobulins are considered an alternative line of therapy, especially in refractory OAPS. Unfortunately, there have been inconsistent results published from randomized placebo-controlled and randomized controlled treatment trials (RCTs) investigating the effect of IVIG on the live birth ratio (LBR) in RPL (recurrent pregnancy loss) patients [81]. Studies have not demonstrated significant reductions in miscarriages or IUGR with the addition of IVIG to LDA and LMWH [82]. However, therapies, including rituximab or pravastatin with immunoglobulin, have shown improvements in live birth rates. The aim of Kaneko’s et al. (2023) study was to compare the efficacy of IVIG therapy for refractory OAPS compared to conventional treatment: 62.5% of cases were able to achieve preferable pregnancy results through this combination [83]. Further research, especially RCTs, into the therapeutic potential in recurrent pregnancies, is warranted, as factors unrelated to OAPS may also be involved [80,84]. A breakthrough therapy for recurrent pregnancy loss in obstetric APS could be treatment with TNF-a blockers. The ongoing IMPACT study will determine the efficacy of certolizumab (which does not cross the placental barrier) in reducing local inflammation and improving placental perfusion [74].

### 4.4. Supplementation

According to recommendations, empiric vitamin D supplementation during pregnancy prevents complications such as preeclampsia, intrauterine mortality, or SGA, suggesting a dosage of 2500 IU/day (range 600–5000 IU/day) [85]. Patients undergoing treatment with heparin or corticosteroids should also receive calcium due to the risk of osteoporosis [86]. Women diagnosed with APS and at average risk of neural tube defects ought to take a minimum dose of 400 μg/day of folic acid during preconception and consider continuing this medication throughout pregnancy [87,88]. Additionally, any modifiable obstetric risk factors, namely smoking, obesity, diabetes, or hypothyroidism, should be closely controlled [86].

## 5. Conclusions

The insufficiency of data research into OAPS patients leads to a lack of standardized protocols, which results in inconsistent care and postponed diagnoses [80]. Appropriate therapy and close monitoring during pregnancy, including clinical observation, placental biomarkers (sFlt-1, PlGF), and regular ultrasonography, may help to reduce the risks and increase chances for optimal pregnancy outcomes. Maternal serum levels of the sFlt-1/PlGF ratio are useful in suspecting complications such as preeclampsia (PE) in higher-risk patients, approximately from the 20th week of gestation onward. Increased levels of placental biomarkers (>38) warrant close monitoring and repeat testing [75].

The timely initiation of therapy with anticoagulants and secondary-line medications, e.g., HCQ, especially in patients with triple aPL positivity, can reduce the risk of adverse obstetric events in APS patients.

The pathological examination of the placenta may also help to better understand the pathogenesis of OAPS and find new effective therapies. Clinical collaboration provided by rheumatologists, obstetricians, and pathologists is an essential component of successful care [74].

## Figures and Tables

**Table 1:** ACR/EULAR 2023 classification criteria for APS.

Entry criteria	At least one positive result of a test for aPLs (LA, a moderate/high titer of ACA or aβ2GPI-class IgG or IgM) within 3 years from the time of the diagnosis of a clinical criterion. or Citeria are listed below in domains 1–6: Venous thromboembolism; Arterial thrombosis; Microvascular; Obstetric complications; Heart valve disease; Thrombocytopenia.
Additional criteria (scored from 1 to 7)	Six clinical domains and two laboratory domains with the presence of the following: LA and ACA and/or aβ2GPI-class IgM or IgG

**Table 2:** ACR/EULAR APS classification criteria- definitions of PreE and PI, with severe features [2,19,20].

PreE with severe features Preeclampsia is a pregnancy-related disorder characterized by new-onset hypertension, typically occurring after the 20th week of gestation and most commonly near term.
Accordingly, this is accompanied by proteinuria or remains as gestational hypertension alone with ≥1 of the following: Blood pressure ≥ 160 mmHg/≥110 mmHg on two occasions; Visual disturbances; Abdominal pain and elevated liver enzymes; Thrombocytopenia (<100 × 10^9^/L); Renal insufficiency; Pulmonary edema.
Placental insufficiency with severe features Placental insufficiency is associated with preeclampsia and intrauterine growth restriction (IUGR), which together lead to preterm labor and increased perinatal morbidity. The 2023 ACR/EULAR APS classification criteria defined IUGR as an estimated fetal weight or postnatal weight < 10th percentile for gestational age with lack of fetal-neonatal syndromes or genetic conditions correlated with growth restriction, including ≥1 of the following: Abnormal or non-reassuring fetal surveillance test(s) suggestive of fetal hypoxemia (e.g., a nonreactive non-stress test); Abnormal Doppler flow velocimetry waveform analysis suggestive of fetal hypoxemia (e.g., absent end-diastolic flow in the umbilical artery); Severe intrauterine fetal growth restriction (in USG) indicating an estimated fetal or postnatal birth weight < 3rd percentile for gestational age; Oligohydramnios (e.g., an amniotic fluid index ≤ 5 cm or deepest vertical pocket < 2 cm); Placental pathology: maternal vascular malperfusion (infarcts, thrombosis, and decidual vasculopathy)

**Table 3:** The suggested management of obstetric APS in non-pregnant and pregnant women [64,69,74,75].

	Preconception	During Pregnancy	
A high-risk profile of aPLs and no history of pregnancy complications	LDA (75–100 mg/day) at least 4 weeks before gestation	LDA * (75–100 mg/day)	(1) Prenatal screening between 11 and 13 weeks + 6 days of gestation with Doppler evaluation of uterine blood flow (UtA), mean arterial pressure (MAP) and the detection of placental growth factors (PlGFs) in the blood. (2) Patients with high-risk conditions ****: prenatal screening between 19 and 24 weeks of gestation must be considered in light of the patient’s history and the evaluation of the uterine artery pulsation index (UtA PI), MAP, PlGF, and sFlt-1 (soluble fms-like tyrosine kinase 1) measurements. The measurement of the plasma/serum sFlt-1/PlGF ratio test measured: Between 20 and 36 weeks + 6 days of gestation—short-term predictor of preeclampsia occurrence; After 37 weeks of gestation, a follow-up was conducted to detect uteroplacental insufficiency; - Asymptomatic: tested monthly from 20 weeks of gestation onwards; - Symptomatic: ratio test 38–85→ monitoring after 1–2 weeks or as soon as the clinical situation changes. Ratio test > 85 (or >110 after 34 w.)→intensive monitoring. (3) Screening for PE at 30–38th week of gestation *****
A history of ≥3 recurrent miscarriages for <10 weeks of gestation or ≥1 fetal loss for ≥10 weeks of gestation		LDA (75–100 mg/day) and heparin at prophylactic dose—LMWH preferred (0.4–0.6 mg/kg/d)	
History of delivery prior to 34 weeks of gestation due to eclampsia/severe preeclampsia/features of placental insufficiency		LDA (75–100 mg/d) or LDA (75–100 mg/d) and heparin at prophylactic dosage	
“Non-criteria” obstetric APS		LDA (75–100 mg/d) or LDA (75–100 mg/d) with heparin based on risk profile	
Pregnancy complications despite combined LDA and heparin at prophylactic dosage treatment		Heparin at a therapeutic dose or with hydroxychloroquine, low-dose prednisolone (10–15 mg/d), or IVIG added in selected cases	
The history of thrombotic APS	VKA **	LDA and heparin at therapeutic dosage (1 mg/kg/day) or with hydroxychloroquine added ***	

* Many recommendations include aspirin doses of 75 to 150 mg/d starting ≥ 12 weeks of gestation to decrease the risk of preeclampsia. ** must be excluded, especially between weeks 5–12 of gestations. *** American guidelines [71]. **** group at a high risk of FGR and/or preeclampsia. ***** Repeat screening for PE at 30–38 weeks, including MAP, UtA PI, serum PLGF, and sFLT-1 (using The Fetal Medicine Foundation (FMF) combined algorithm similar to previous measurements). In the case of thrombocytopenia (≤20,000 platelets), stop LDA and reduce the dose of LMWH to 0.2 mg/kg/day. IVIG—intravenous immunoglobulin. LDA—low-dose aspirin. LMWH—low molecular weight heparin. UFH—unfractionated heparin. VKA—vitamin K antagonists.

## Data Availability

Data are available on request due to restrictions as the data contains personal and clinical information that is subject to privacy, legal, and ethical constraints.

## Abbreviation

ACOG—American College of Obstetricians and Gynecologists; ACA—anticardiolipin antibodies; aCL—anticardiolipin antibody; AIDs—autoimmune diseases; ANA—anti nuclear antibody; aPLs—antiphospholipid antibodies; ApoER2—apolipoprotein E receptor 2; APS—antiphospholipid syndrome; ASA—acetylsalicylic acid; aβ2GPI—anti-β2-glycoprotein I antibody; β2G PI—phospholipid-binding proteins; CAPS—catastrophic APS; COC—combined oral contraceptive; CPR—cerebroplacental ratio; EBV—Epstein–Barr-virus; EFW—estimated fetal weight; EPCR—endothelial protein C receptor; EVTs—extravillous trophoblasts; FGR—fetal growth restriction; FVM—fetal vascular malperfusion; HBV—hepatitis B virus; HCG—human chorionic gonadotropin; HCQ—hydroxychloroquine; HCV—hepatitis C virus; HIV—human immunodeficiency virus; HLA—human leukocyte antigens; HTLV-1—human T-cell leukemia type 1; IFN-alpha—interferon alpha; IL-6—interleukin 6; IL-8—interleukin 8; ISSHP—The International Society for the Study of Hypertension; IUGR—intrauterine growth restriction; IVIG—intravenous immunoglobulins; LA—lupus anticoagulant; LDA—low-dose aspirin; LMWH—low-molecular-weight heparin; MAP—mean arterial pressure; MVM—maternal vascular malperfusion; NC-OAPS—non-criteria obstetric APS; OAPS—obstetric antiphospholipid syndrome; PE—preeclampsia; PI—pulsatility index; PlGF—placental growth factor; sFlt-1—soluble fms-like tyrosine kinase 1; sFlt/PIGF—soluble fms-like tyrosine kinase 1 and placental growth factors index; SGA—small-for-gestational-age; SLE—systemic lupus erythematosus; SNAs—syncytial nuclear aggregates; TF—tissue factor; TLR4—toll-like receptor 4; TNF-alpha I—tumor necrosis factor α; TORCH—toxoplasmosis, other, rubella, cytomegalovirus, herpes simplex virus; UFH—unfractionated heparin; UtA-PI—uterine artery pulsatility index; VEGF—vascular endothelial growth factor; VSM—vasculosyncytial membranes.
